# Feasibility of 3D-Printed PLA Meshes in Gypsum Composites: Preliminary Experiments and Insights

**DOI:** 10.3390/polym17111562

**Published:** 2025-06-04

**Authors:** Ahmet Hayrullah Sevinç, Muhammed Yasin Durgun

**Affiliations:** 1 Construction Programme, Kahramanmaraş İstiklal University, 46340 Elbistan, Turkey; 2 Department of Civil Engineering, Bartın University, 74110 Bartın, Turkey; mydurgun@bartin.edu.tr

**Keywords:** gypsum composites, 3D-printing, PLA fibers, fiber mesh, engineering properties

## Abstract

The mechanical limitations of gypsum-based composites necessitate reinforcement strategies to enhance their structural performance. This study investigates the feasibility of integrating 3D-printed polylactic acid (PLA) meshes into gypsum composites through a series of preliminary experiments. Various mesh configurations were tested, including different fiber thicknesses, mesh grid sizes, and single- and double-layer applications. The impact of mesh incorporation on bulk density, ultrasonic pulse velocity (UPV), bending strength, and compressive strength was assessed. The results indicate that the inclusion of PLA meshes had a limited effect on bulk density and led to a slight decrease in UPV values, suggesting increased porosity. Although improvements in mechanical properties were anticipated, most specimens exhibited lower bending and compressive strengths compared to the reference specimen. Among the tested configurations, 2 mm thick meshes demonstrated relatively higher performance, particularly in bending strength, with narrow-mesh aperture yielding better results. However, double-layer mesh applications consistently resulted in lower strength values. These findings highlight the challenges associated with integrating 3D-printed PLA meshes into gypsum composites. While the study provides valuable insights into mesh-based reinforcement, further investigations are required to optimize fiber–matrix interactions and enhance mechanical performance. Future research should explore alternative printing parameters, improved adhesion techniques, and hybrid reinforcement approaches to fully exploit the potential of additive manufacturing in gypsum-based composites.

## 1. Introduction

Gypsum has been known as a construction material since ancient times, spanning nearly 4000 years. Today, it remains widely used in construction due to its numerous advantages, including low cost, lightweight nature, excellent thermal and acoustic insulation properties, and resistance to fire. However, gypsum is a material with limited mechanical properties [1,2]. It is well known that ettringite-based binders exhibit a brittle nature [3]. Consequently, gypsum is unable to withstand various loads and impacts encountered in structural applications effectively. Even relatively minor impacts can cause damage to gypsum, which may negatively affect its other advantageous properties [1].

Reinforcing gypsum with fibers has proven to be an effective solution to address these limitations. In fiber-reinforced gypsum-based systems, the bridging effect of the fibers enhances toughness [3].

In cement matrices, gypsum matrices, or other applications, reinforcement is often employed to improve mechanical properties through fibers. This reinforcement can be achieved by homogeneously distributing short fibers within the matrix. Alternatively, fibers can also be applied in the form of meshes, grids, or sheets, offering a different approach to enhance the material′s performance. Although short fibers are considered effective in improving the properties of materials, they are often deemed insufficient for reinforcing structural components. For this reason, mesh fabrics composed of basalt, glass, or carbon fibers are commonly employed in certain structural applications. Flexible meshes can be used as adhesives or wraps; however, they are not suitable for layered production. Conversely, stiffer mesh structures can be utilized for layered manufacturing [4]. Furthermore, the literature indicates that using continuous fiber meshes instead of randomly distributed short fibers is more successful in achieving the desired performance characteristics. This is particularly significant in cases where a high volume of fibers is required, as continuous fiber meshes offer superior results [5].

Liu et al. utilized steel fiber meshes in different layers within 3D-printed concrete in their study. As a result, they were able to alter the fracture modes of the structure from brittle to more ductile behavior. It was reported that the deformation capacity increased by a factor of 11 [4]. Wang et al. investigated the use of fiberglass mesh and short glass fibers separately and in hybrid form in epoxy synthetic foams. The results indicated that fiberglass mesh was found to be more effective than short glass fibers. Additionally, the number of mesh layers and their placement were identified as critical parameters influencing performance [5]. In another study, epoxy-coated nylon threads and glass fiber mesh were used in sandwich concrete structures. The results indicated that while the implemented systems did not enhance flexural strength, they effectively altered the fracture characteristics from brittle to ductile behavior [6]. In a study measuring the impact resistance of gypsum boards, samples reinforced with mesh, wallpaper, and a combination of both were subjected to impact tests. The results reported that the use of 75 g/m^2^ mesh reinforcement increased impact resistance by 58% [7]. Chen et al. investigated the reinforcement of foamed lightweight soil using short glass fibers, glass fiber mesh, and their hybrid combination. The results showed that the flexural strength of the produced materials increased by at least 63% with the use of short fibers alone, while the use of fiber mesh led to a minimum increase of 202%. Fiber mesh was found to have a significant effect on flexural strength. Additionally, the hybrid use of fiber mesh and short fibers further enhanced this increase to 278% [8]. In another study, polyvinyl alcohol and polypropylene fibers, along with wire meshes of various sizes, were used in thin sheet mortars. The results indicated that in extremely thin mortar layers, steel wires in mesh form influenced the maximum strength. Additionally, the use of short fibers contributed to crack control and helped fill the gaps between the mesh layers [9]. In a study utilizing hessian fiber meshes in different configurations with alpha and beta gypsum, one-, two-, and three-layer hessian mesh variants were tested. However, all these configurations exhibited lower flexural strength compared to the reference samples. On the other hand, the sample reinforced with two layers of fiberglass mat demonstrated the highest strength. The findings highlighted the critical importance of the bond between the mesh fibers and the matrix. Although hessian fiber meshes did not enhance flexural strength, they were observed to result in a more gradual failure mode [10]. Shamseldein et al. investigated the use of basalt fiber meshes with different grid sizes in multiple layers. Their study found that in conventional mortars, the use of basalt fiber meshes increased strength up to four layers. Additionally, while an increase in mesh grid size from 5 mm to 10 mm generally resulted in a slight decrease in strength, a further increase to 34 mm led to an improvement in strength [11]. In his study, Pablo produced meshes using natural fibers, specifically Abaca and Sisal, and incorporated them into cementitious mortars. The effect of fiber layers arranged in two, four, and six layers on flexural strength was examined. The results indicated that an increase in the number of layers enhanced flexural strength. The use of Abaca fiber mesh led to strength improvements of up to 85%, while Sisal fiber mesh resulted in increases of up to 74%. Additionally, an analysis of the toughness modulus revealed increases of up to 400% and 353%, respectively [12]. Zhang et al. utilized textile-reinforced geopolymer mortars as reinforcement elements in concrete slabs. Carbon fiber mesh was selected as the textile material. The flexural strength of reinforced concrete slabs increased by 26%, 53%, and 92% with the use of one, two, and three layers of mesh, respectively. It was concluded that this reinforcement approach is effective in enhancing flexural capacity and maintaining post-crack stiffness [13]. In another study, cementitious composites reinforced with AR-glass fiber mesh and polypropylene fiber mesh were compared. It was observed that the mechanical properties of polypropylene fiber mesh were lower than those of AR-glass fiber mesh, leading to the application of adhesion-enhancing treatments to the polypropylene fibers. As a result, the flexural strength of specimens reinforced with polypropylene fiber mesh improved, and notably, the first crack resistance was reported to be even higher than that of the AR-glass fiber mesh [14]. In their study, Wang et al. investigated the use of steel fiber mesh with two different grid sizes and Kevlar fiber mesh in cementitious composites. These reinforcements were applied in various layer configurations, including fiber-free, polyethylene fiber-reinforced, and polyvinyl alcohol fiber-reinforced composites. An analysis of the modulus of rupture (MOR) values revealed that an increase in fiber volume enhanced strength and reduced average crack spacing. The study concluded that the best performance could be achieved with the use of Kevlar fibers in two layers combined with 1.5% polyethylene fibers [15]. Shi et al. utilized woven glass fiber mesh to enhance the resistance of polyurea coatings under explosive loading. The results indicated that the use of woven glass fiber mesh altered the failure mode of polyurea coatings from shear punching to tensile failure [16]. Boccarusso et al. investigated the use of hemp fiber mesh with two different areal densities in gypsum matrices. They examined the effects of mesh placement and layer count within the composite, as well as the impact of partial saturation of the fibers with bio-epoxy resin. The highest tensile stress values were obtained from specimens incorporating high areal density fibers saturated with epoxy. Additionally, it was reported that a two-layer configuration, with one mesh placed at the top and one at the bottom, was more effective than a single-layer mesh placed in the middle [17].

As seen in the literature, while there are several studies comparing the use of fibers and meshes in cementitious composites and examining the effects of their hybrid applications on the final product, such studies remain quite limited for gypsum-based composites. In this study, mesh production was carried out using 3D printing technology to integrate the reinforcement of gypsum matrix materials with additive manufacturing. As is well known, 3D printing technology is a form of additive manufacturing. It involves the transformation of a digitally designed model into a physical object by printing it layer by layer using appropriate materials [18]. First introduced in 1986, this technology has continuously evolved and has become widely adopted. By 2016, 3D printing had gained significant popularity worldwide and was extensively used in various fields such as automotive design, rapid prototyping, aerospace, defense, architectural design and modeling, and healthcare.

In parallel, research has intensified on both 3D printing techniques and 3D printing materials. As a result, fundamental techniques such as Fused Deposition Modelling (FDM), Stereolithography (SLA), and Selective Laser Sintering (SLS) have been developed. Along with these techniques, various materials, including polylactide (PLA), acrylonitrile-butadiene-styrene (ABS), polycarbonate (PC), and polyamide (PA), have been introduced as 3D printing materials.

There are few studies in the literature on the use of fibers produced with 3D printing technology in construction materials. However, an example in this field is the study by Santana et al., which explores the printing and application of 3D-printed mesh in geopolymer mixtures [19].

Although materials such as glass and carbon fibers have found their place in 3D printing technologies, PLA remains the most widely used filament type today. Moreover, glass and carbon fibers are already commercially available in the construction materials industry and have been evaluated in numerous studies. The main reason for selecting PLA in this study is to explore a material that has not previously been used in construction applications but is well-integrated into 3D printing technologies. In this study, various mesh structures were produced using polylactide (PLA), one of the most commonly used filaments in 3D printing technology. As this research serves as a preliminary investigation, parameters such as fiber thickness, mesh grid size, and layer count, which can be reliably manufactured using 3D printing, were examined. The study focused on determining the most suitable mesh configuration for gypsum-based matrices and its effective application.

## 2. Materials and Methods

### 2.1. Materials

**Figure 1:**
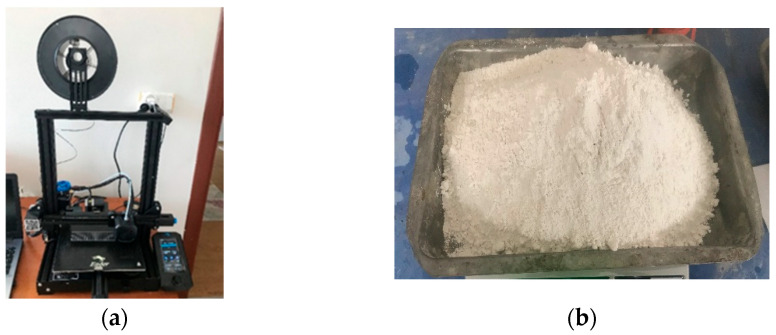
(**a**) 3D-Printer; (**b**) Gypsum.

The gypsum used in this study was commercially obtained (ABS Gypsum, İstanbul, Turkiye) and complies with the TS EN 13279-1 standard [20]. The technical properties of the gypsum are provided in Table 1. Polylactic acid (PLA) filament (Tinylab 3D, İstanbul, Turkiye) was used for the production of the 3D meshes, and its technical properties are presented in Table 2. The 3D printer (Ender-3 V2 model, Shenzhen, China) and the gypsum used in this study are shown in Figure 1.

### 2.2. Methods

Since the objective of this study was to determine the most suitable mesh type using a 3D printer (which has a nozzle diameter 0.4 mm, layer thickness 0.16 mm and 100% infill), preliminary trials were conducted by varying parameters such as thickness, aperture size, and layer count. Based on the results obtained, the weaknesses of the selected parameters were evaluated, leading to further trials with new parameter adjustments to optimize the mesh fiber type.

In each preliminary trial phase, the produced mesh fibers were placed inside prismatic molds measuring 40 × 40 × 160 mm, followed by the casting of gypsum. The fresh gypsum paste was prepared using 1200 g of gypsum powder and 720 g of water, resulting in a water-to-gypsum ratio of 0.60.

For single-layer mesh applications, gypsum was poured halfway into the mold, and the mesh was placed and fixed at the marked center point before completing the mold filling with gypsum. In double-layer mesh applications, a similar layered production approach was adopted, with the meshes positioned 0.5 cm from both the top and bottom of the mold.

**Figure 2:**
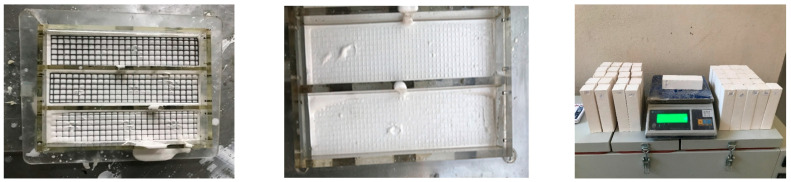

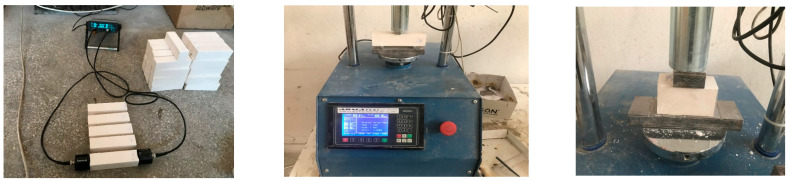
Images of experimental procedures.

The specimens were demolded after 60 min and stored in laboratory conditions for 7 days. Before testing, they were placed in an oven at 60 °C for 48 h to remove free moisture. Although PLA has a softening point in the range of 60–65 °C, it is important to note that in this study, the meshes were completely embedded within the gypsum composite. Given gypsum’s known fire-resistance and low thermal conductivity, it is unlikely that the PLA meshes experienced critical thermal exposure during the 60 °C curing process. The specimens were then subjected to bulk density testing (ASTM C 138) [21], ultrasonic pulse velocity (UPV) testing (ASTM C 597) [22], and bending and compressive strength tests (TS EN 196-1) [23]. Images related to the tests are presented in Figure 2.

## 3. Results and Discussion

### 3.1. First Preliminary Trials

**Figure 3:**
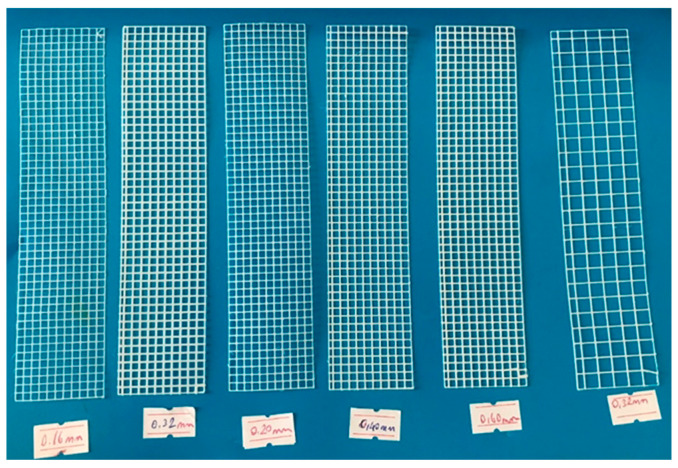
Fiber meshes used for first preliminary study.

For the first preliminary trials, fiber thicknesses of 0.16 mm, 0.20 mm, 0.32 mm, 0.40 mm, and 0.60 mm were selected. Narrow square apertures of 3 × 3 mm were chosen for some samples, while wide square apertures of 6 × 6 mm were used for others. The meshes produced with thicknesses of 0.16 mm, 0.20 mm, and 0.32 mm were tested in both single-layer and double-layer applications. In this initial trial phase, selecting extremely thin fiber thicknesses posed challenges in mesh production. Some defective fibers were produced before achieving structurally sound meshes suitable for application in gypsum composites. The production details and corresponding sample codes for this preliminary trial are listed in Table 3, while the produced fibers are shown in Figure 3.

**Figure 4:**
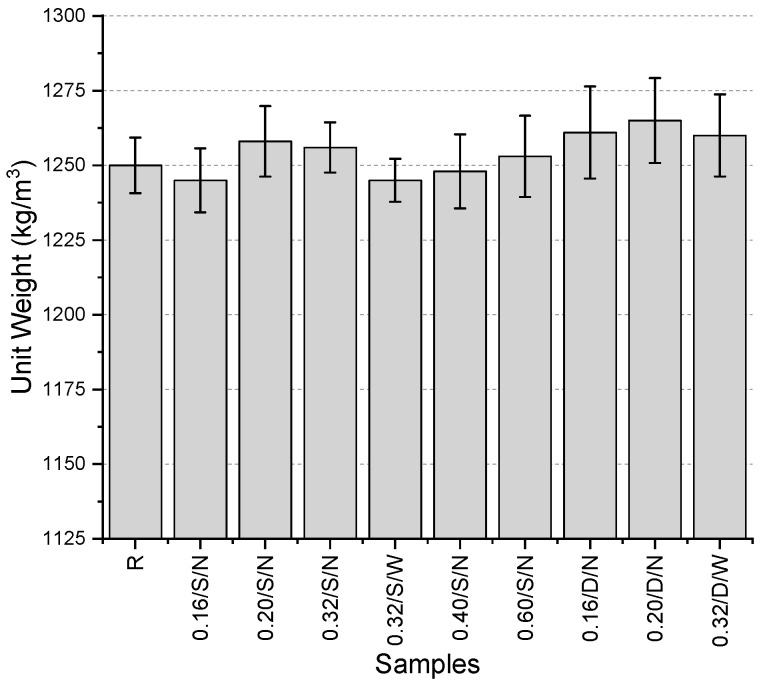
Unit weights of first preliminary trials.

The unit weight values (Figure 4) of the samples produced in the initial trials ranged between 1245 and 1265 kg/m^3^. It was observed that the values varied within a narrow range, with only a 1.6% difference between the highest and lowest values. The lowest values were obtained using a single-layer, narrow-aperture mesh with a fiber thickness of 0.16 mm and single-layer, wide-aperture mesh with a fiber thickness of 0.32 mm, while the highest value was recorded for a double-layer, narrow-aperture mesh with a fiber thickness of 0.20 mm. When evaluating single-layer mesh samples separately, the highest unit weight was also observed in the sample using a 0.20 mm thick narrow-aperture mesh. Naturally, the use of the same fibers in a double-layer configuration resulted in an increase in unit weight. Additionally, for the 0.32 mm thick fibers, both narrow and wide aperture meshes were tested, and it was found that using a wide-aperture mesh led to an increase in unit weight. No significant trend was observed in the variations related to fiber thickness.

**Figure 5:**
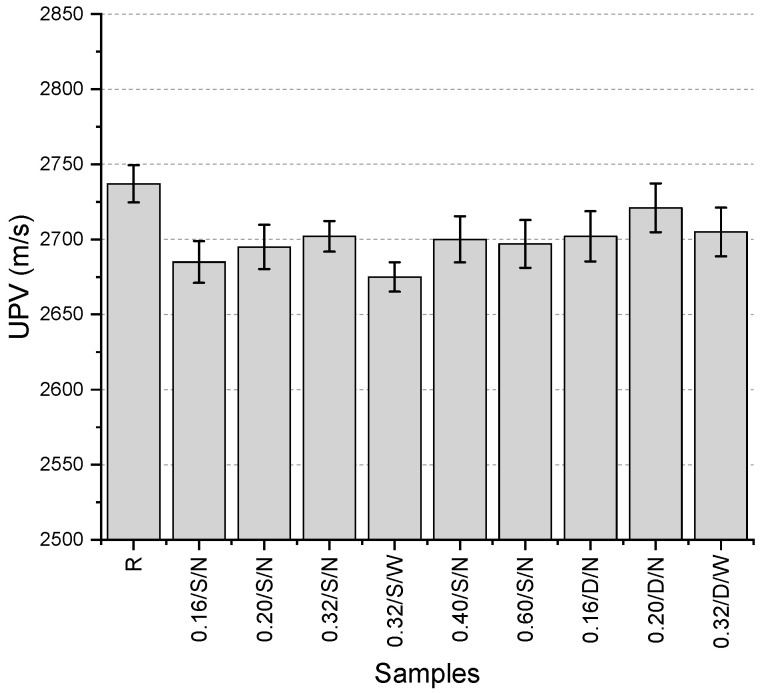
UPV values of first preliminary trials.

The UPV values (Figure 5) range between 2675 and 2737 m/s, with the highest value obtained from the reference specimen. The UPV test is influenced by voids and cracks within the material structure. A more porous structure or the presence of cracks leads to a decrease in UPV values [24]. The fact that the highest value was obtained from the reference specimen suggests that minor casting issues may have occurred due to the use of pre-prepared fibers, potentially resulting in slight void formation. The reason for considering the void formation to be minimal is the narrow distribution of UPV values, as the difference between the highest and lowest values is only 2.3%. The lowest value was obtained from the single-layer mesh specimen with a fiber thickness of 0.32 mm and a wide mesh aperture. Among the specimens containing mesh, the highest value was recorded for the 0.20/D/N specimen (2721 m/s). For single-layer mesh specimens, the highest value was observed in the 0.32/S/N specimen. When comparing single-layer and double-layer mesh configurations for the same specimen, the double-layer mesh provided higher results. Additionally, specimens with the same mesh layer and fiber thickness, those with a narrower mesh aperture exhibited slightly higher UPV values.

**Figure 6:**
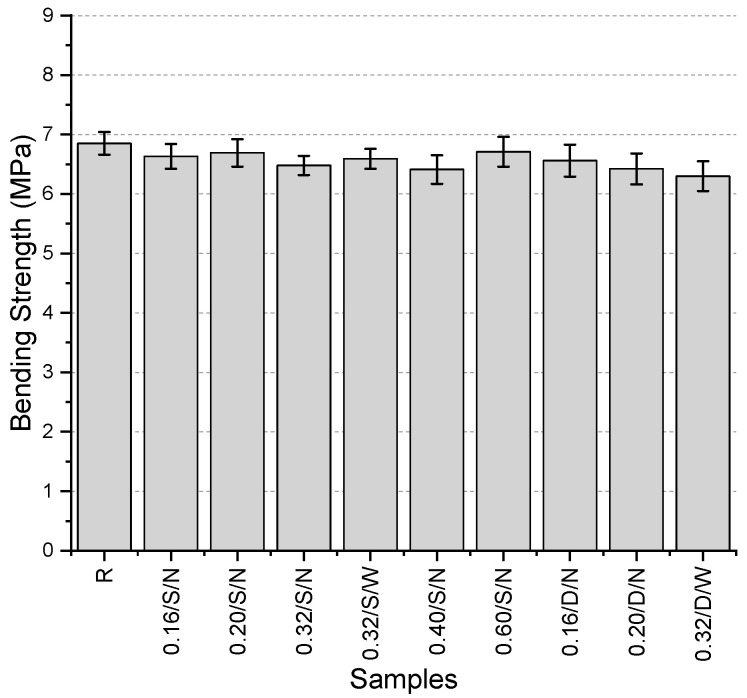
Bending strength values of first preliminary trials.

The bending strength values (Figure 6) range between 6.3 and 6.85 MPa, with the highest value obtained from the reference specimen. Although an increase in bending strength was expected with the use of mesh fibers, all values were found to be lower than the reference. However, the maximum reduction observed compared to the reference was only 8%. Among the specimens containing mesh fibers, the highest bending strength was recorded for the 0.60/S/N specimen, with a value of 6.71 MPa. This finding suggests the necessity of increasing fiber thickness. When examining the single- and double-layer mesh versions of the specimens with fiber thicknesses of 0.16 mm and 0.20 mm, it was observed that the double-layer mesh specimens exhibited higher bending strength than their single-layer counterparts. This increase was 1.1% and 4%, respectively. Additionally, in the specimen with a fiber thickness of 0.32 mm and a single-layer mesh, the use of a wide mesh aperture resulted in a 1.7% increase in bending strength. Among all specimens, the lowest bending strength was observed in the specimen produced with a double-layer mesh with a wide mesh aperture and a fiber thickness of 0.32 mm.

**Figure 7:**
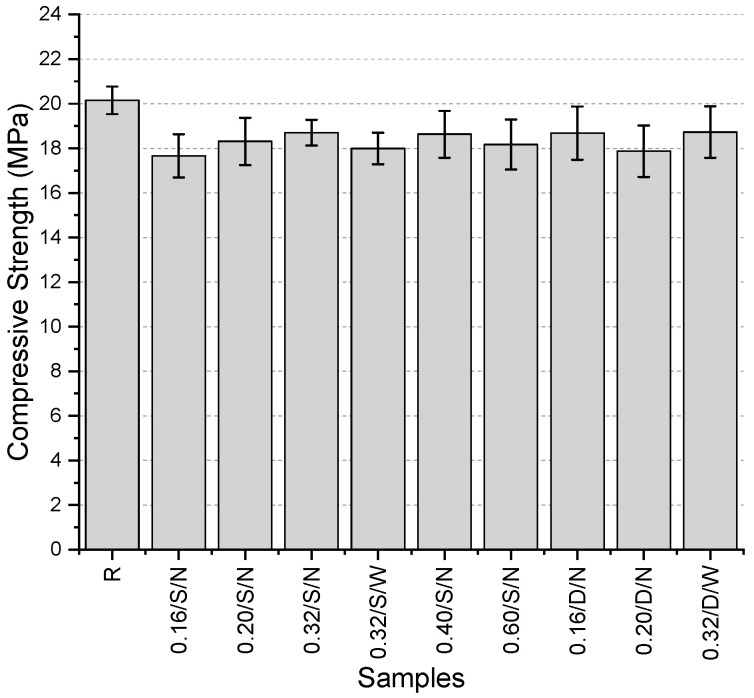
Compressive strength values of first preliminary trials.

The compressive strength values (Figure 7) of the specimens’ range between 17.7 and 20.2 MPa, with the highest value obtained from the reference specimen. Among the specimens with a single-layer narrow-mesh configuration, the highest compressive strength was recorded in the specimen containing 0.40 mm fibers, with a value of 18.6 MPa. For the specimens containing 0.32 mm thick fibers, where the mesh aperture was varied between narrow and wide, an increase in mesh aperture resulted in a 3.8% reduction in compressive strength. Additionally, in specimens with the same fiber thickness and wide mesh aperture, the use of a double-layer mesh increased compressive strength by 4.1% compared to the single-layer configuration. Among all specimens, the lowest compressive strength was observed in the 0.16/S/N specimen.

**Figure 8:**
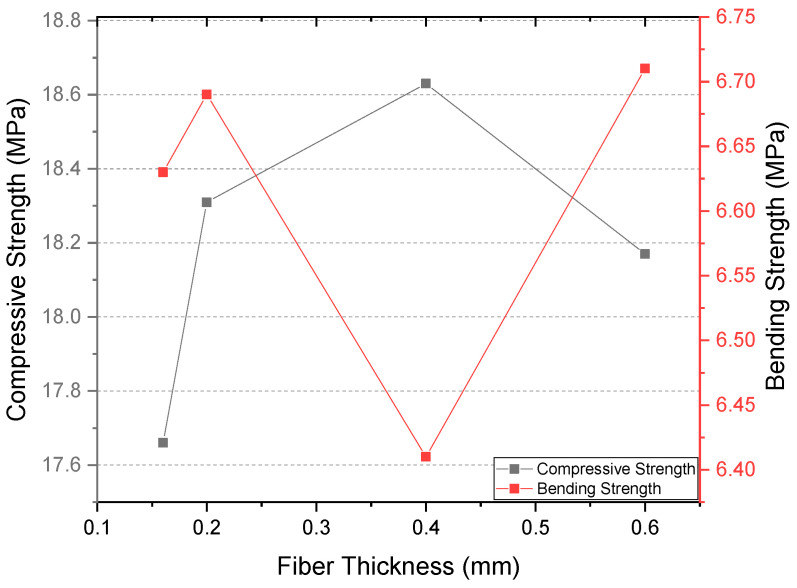
Effect of fiber thickness on mechanical performance under identical S-N mesh configuration.

An extended comparison under single-layer, narrow-aperture (S–N) mesh configuration revealed that fiber thickness has a non-linear effect on mechanical properties (Table 4). Compressive strength increased from 17.66 MPa (0.16 mm) to a peak of 18.63 MPa (0.40 mm), but slightly declined at 0.60 mm (18.17 MPa), suggesting a saturation point in reinforcement efficiency (Figure 8). Interestingly, flexural strength showed its maximum at 0.60 mm (6.71 MPa), possibly due to enhanced crack-bridging capabilities with thicker fibers. These results indicate that the optimal fiber thickness may depend on the specific mechanical performance criteria being targeted.

When all results of the produced specimens are evaluated, it is observed that the use of mesh did not have a significant impact on the outcomes. In none of the trials was any improvement observed in the mechanical properties, which were expected to be enhanced. On the contrary, albeit by small margins, the specimens yielded lower values compared to the reference specimen. Therefore, modifications were made to the parameters and combinations, leading to the initiation of the second preliminary testing phase.

### 3.2. Second Preliminary Trials

**Figure 9:**
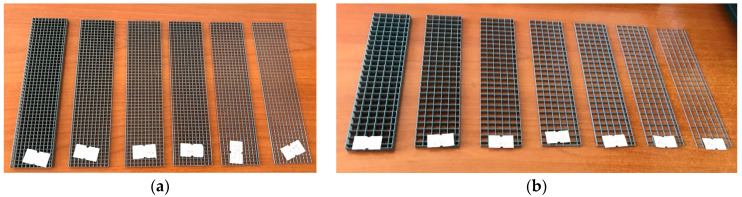
Fiber meshes used for the second preliminary trials: (**a**) Narrow aperture meshes; (**b**) Wide aperture meshes.

In the first trial productions, the low bending strength values, frequent production issues due to the thin fiber thickness, and brittle fractures observed in the matrix due to the inability of the meshes to provide significant reinforcement led to an effort to increase fiber thickness. In this context, the minimum thickness was set at 1.0 mm and the maximum at 5.0 mm, with selected thicknesses of 1.0, 1.5, 2.0, 2.5, 3.0, 4.0, and 5.0 mm. As in the first preliminary trials, the aperture geometries were square, with two different sizes: narrow and wide. To isolate the effect of thickness, all mixtures in this phase used a single-layer mesh. No production defects occurred at these thickness levels. Images of the produced meshes are presented in Figure 9, while the sample codes and characteristics of the specimens are provided in Table 5.

**Figure 10:**
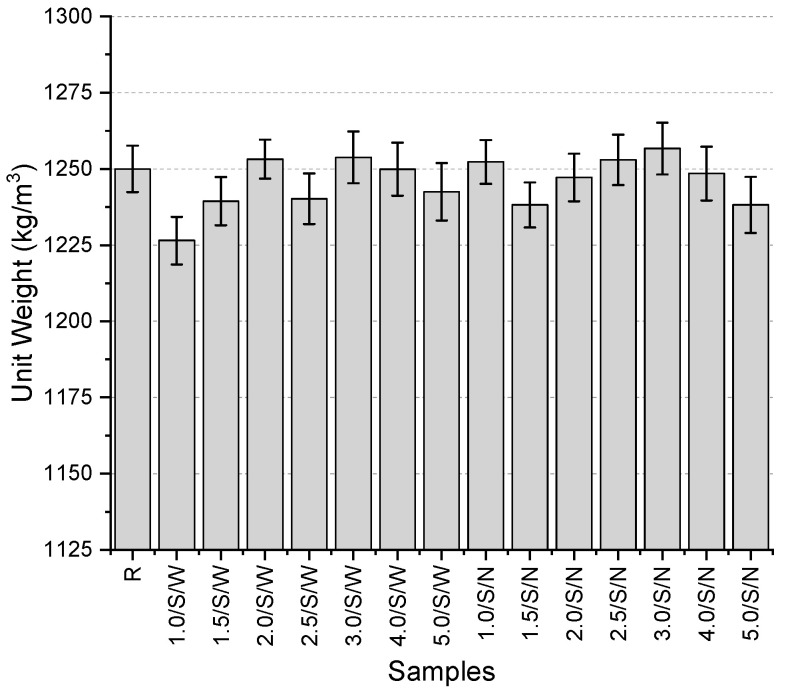
Unit weights of second preliminary trials.

The results of the unit weight tests (Figure 10) range from 1238.2 to 1256.7 kg/m^3^. The reference specimen without mesh has a unit weight of 1250 kg/m^3^. The unit weight values are distributed within a very narrow range in the second preliminary testing phase as well. Compared to the reference specimen, a maximum decrease of 0.9% and an increase of 0.5% were observed. In other words, the change relative to the reference specimen did not even reach 1%. The variation in unit weights with the addition of mesh did not follow a specific trend; however, specimens with 3 mm fiber thickness, both with narrow and wide mesh aperture, provided the highest values with small differences. The resulting table indicates that the unit weight values were not significantly affected by the use of mesh.

**Figure 11:**
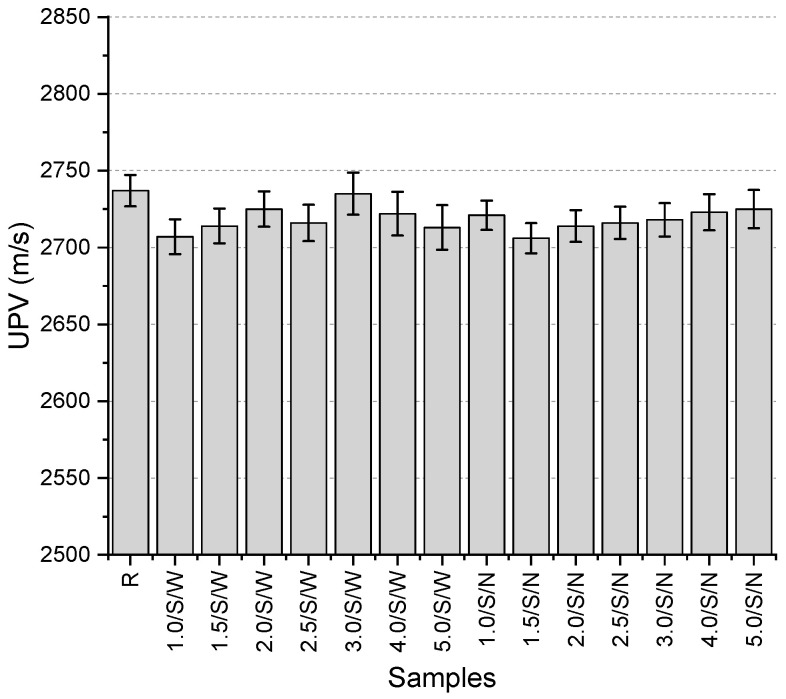
UPV values of second preliminary trials.

The UPV values of the specimens (Figure 11) range from 2706 to 2737 m/s. The highest value was obtained from the reference specimen. In the specimens with a mesh having a wide mesh aperture, the lowest value was obtained from the sample with 1 mm thick mesh, while in the specimens with a narrow mesh aperture, the sample with 1.5 mm thick mesh provided the lowest value. The highest values, on the other hand, were obtained from the samples with 5 mm and 3 mm thick mesh, respectively, for narrow and wide mesh aperture. No clear relationship could be established between the UPV values and whether the specimens had narrow or wide mesh aperture. In some cases, the specimens with narrow mesh aperture gave higher values, while in other cases, specimens with wide mesh aperture provided higher values with the same thickness. The most significant finding from this test is that the use of mesh resulted in a decrease in UPV values.

**Figure 12:**
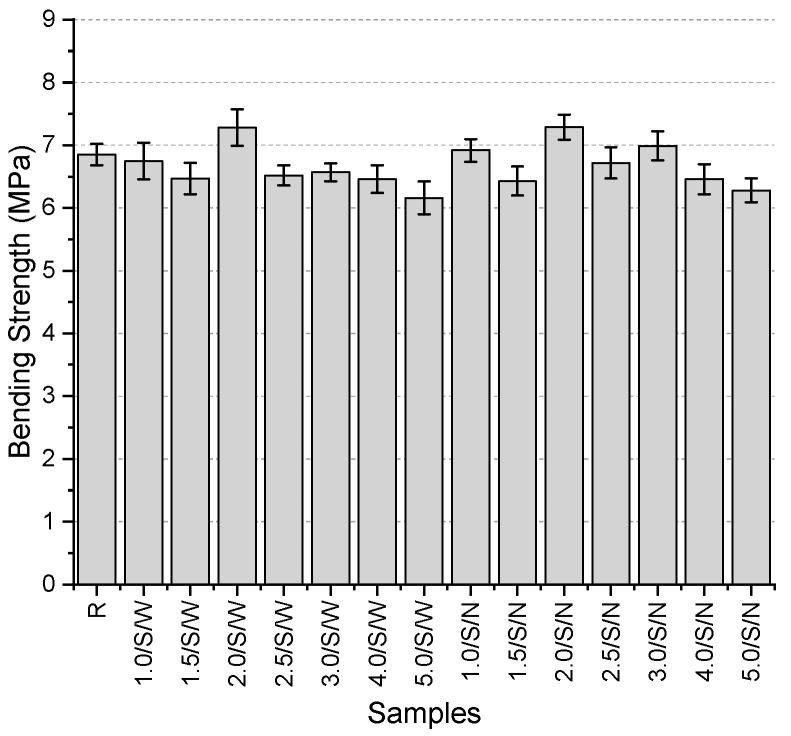
Bending strength values of second preliminary trials.

The bending strength of the specimens (Figure 12) ranges from 6.16 to 7.29 MPa. The reference specimen has a bending strength of 6.85 MPa. It was observed that the majority of the specimens exhibited lower bending strength than the reference specimen. The lowest bending strength was recorded in the 5.0/S/W specimen, which showed a 10.1% lower strength compared to the reference. Although the reduction in bending strength across the specimens did not follow a clear trend, the lowest values among both the wide and narrow mesh aperture specimens were obtained from the series incorporating 5 mm thick meshes. However, no direct relationship could be established between the increase in thickness and strength variation. Among the 14 specimens, only 4 exhibited higher strength values than the reference specimen. These were 1.0/S/N, 3.0/S/N, 2.0/S/W, and 2.0/S/N, with respective increases of 1.0%, 2.0%, 6.3%, and 6.4%. It was observed that higher-than-reference strength values were generally obtained with the use of narrow-mesh specimens. The fact that the best values were obtained from specimens with both narrow and wide mesh aperture using 2 mm thick fibers suggests that this thickness might be an optimal value.

**Figure 13:**
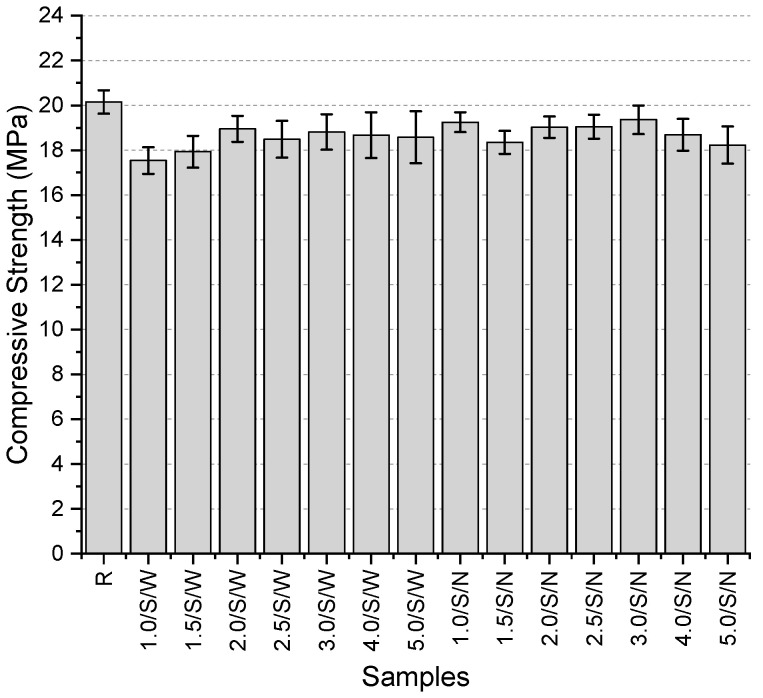
Compressive strength values of second preliminary trials.

The compressive strength values (Figure 13) range from 18.23 to 20.15 MPa, with the highest value obtained from the reference specimen. The use of mesh fibers caused a slight reduction in compressive strength, with a maximum decrease of 9.5%. No clear trend was observed regarding the changes in strength based on fiber thickness. Among the specimens with wide mesh aperture, the lowest compressive strength was recorded for the 1 mm thick mesh, while the highest was obtained for the 2 mm thick mesh. In the specimens with narrow mesh aperture, the highest strength was achieved with the 3 mm thick mesh, whereas the lowest was observed with the 5 mm thick mesh. In all specimens except for the one incorporating a 5 mm thick mesh, the use of narrower mesh aperture resulted in higher compressive strength values. However, the extent of this increase varied between 0.1% and 9.7%.

After this preliminary testing phase, the relatively less successful specimens were eliminated. For the next phase of preliminary testing, a new plan was devised with narrower working ranges, also considering the use of both single and double-layer mesh configurations.

### 3.3. Third Preliminary Trials

**Figure 14:**
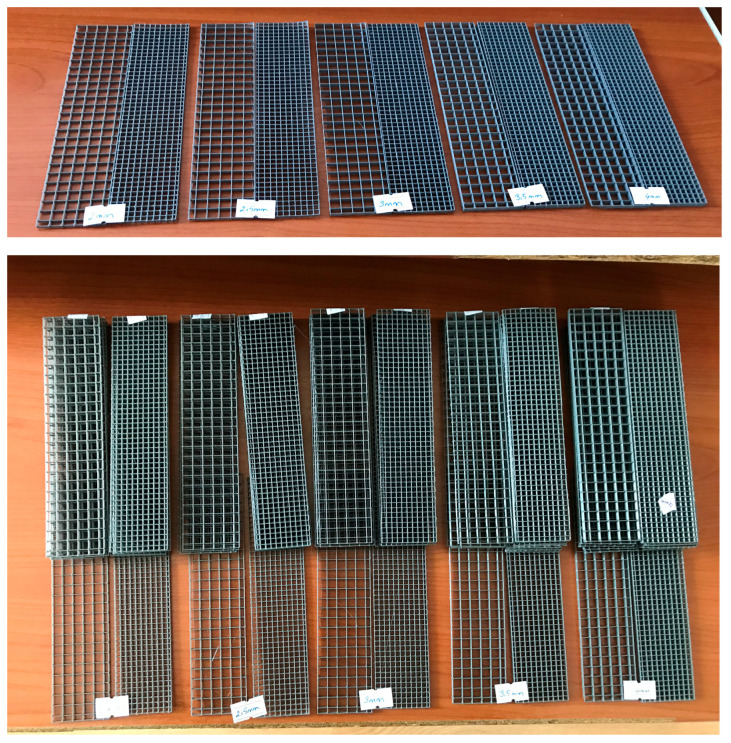
Fiber meshes used for the third preliminary trials.

Upon observing the positive effects of increased thickness in the second preliminary trials, a third trial was conducted, focusing on the most successful results. In this phase, samples were produced using both narrow and wide aperture meshes, as well as in single-layer and double-layer configurations. Meshes with fiber thicknesses of 2.0, 2.5, 3.0, 3.5, and 4.0 mm were used in this stage. Images of the samples are presented in Figure 14, while their characteristics and sample codes are provided in Table 6.

**Figure 15:**
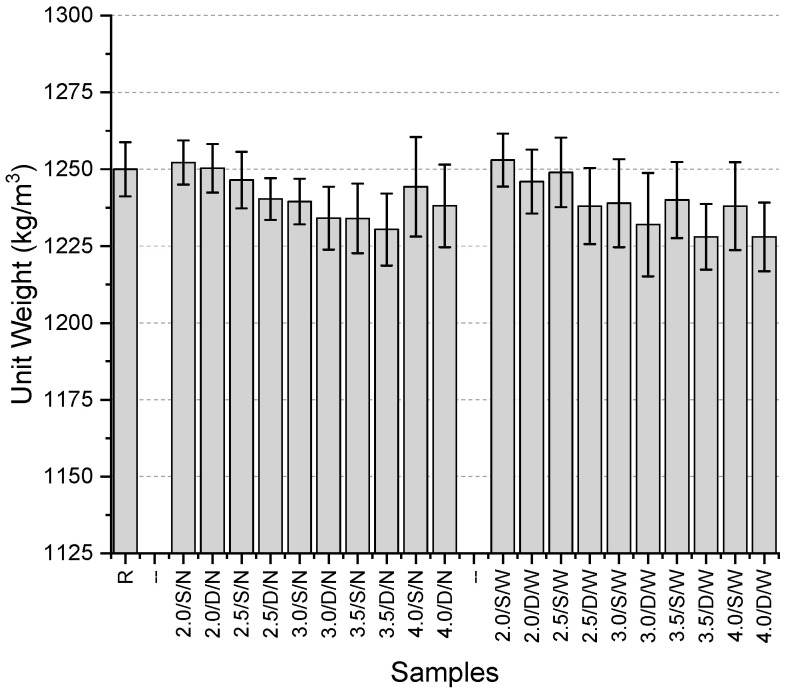
Unit weights of third preliminary trials.

The unit weight values of the specimens (Figure 15) range between 1228 kg/m^3^ and 1253 kg/m^3^. The reference specimen has a unit weight of 1250 kg/m^3^. Among the specimens with a narrow mesh aperture, the highest values were obtained from fibers with a thickness of 2 mm in both single-layer and double-layer mesh configurations (1252.2 kg/m^3^ and 1250.3 kg/m^3^, respectively). The lowest values were recorded for the 3.5/S/N specimen in the single-layer configuration (1234 kg/m^3^) and the 3.5/D/N specimen in the double-layer configuration (1230.4 kg/m^3^). For specimens incorporating a wide mesh aperture, the highest values were again obtained with fibers of 2 mm thickness in both single-layer and double-layer configurations (1253 kg/m^3^ and 1246 kg/m^3^, respectively). The lowest values were observed in the 4.0/S/W specimen for the single-layer configuration and in specimens with 3.5 mm and 4 mm fiber thickness for the double-layer configuration. The maximum decrease compared to the reference was 1.74%, while the maximum increase was 0.24%.

Overall, no clear increasing or decreasing trend was observed, but the use of 2 mm-thick mesh fibers consistently resulted in higher unit weight values. Conversely, the use of 3.5 mm and 4 mm thick mesh fibers led to lower unit weight values. The effect of single-layer versus double-layer mesh configurations on the results was not distinctly evident.

**Figure 16:**
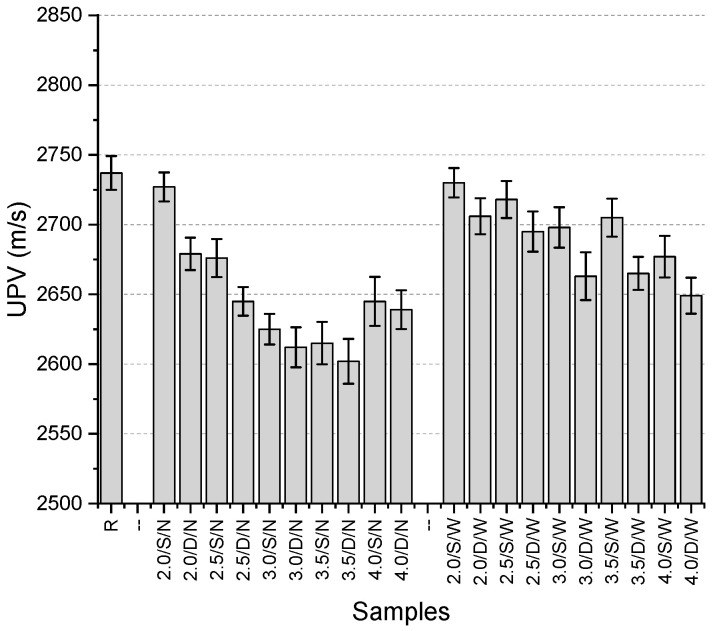
UPV values of third preliminary trials.

The UPV results (Figure 16) range between 2602 and 2737 m/s, with the highest value obtained from the reference specimen. The use of mesh resulted in a reduction in all UPV values. The maximum decrease was 4.9%, indicating that the variations in UPV values were not significant. Among the specimens incorporating a narrow mesh aperture, the highest value was obtained with the use of a single-layer 2 mm thick mesh. Although the UPV value decreased by 1.8% when the same mesh was used in a double-layer configuration, it still yielded the highest value among the double-layer narrow mesh specimens. The lowest values in this series were observed for the 3.5 mm thick fibers in both single-layer and double-layer configurations (2615 m/s and 2602 m/s, respectively).

For specimens with a wide mesh aperture, the highest UPV values were again recorded for the 2 mm fiber thickness in both single-layer and double-layer configurations. The lowest values were obtained with 4 mm thick fibers for both configurations. The general trend observed with increasing mesh aperture was an increase in UPV values. Additionally, the use of double-layer mesh consistently resulted in lower UPV values across all specimens.

**Figure 17:**
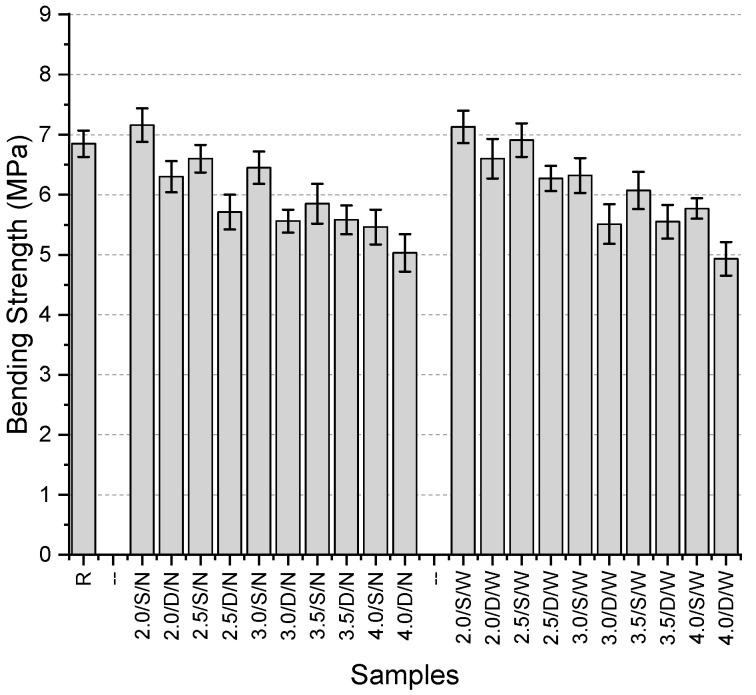
Bending strength values of third preliminary trials.

The bending strengths of the specimens (Figure 17) range from 4.93 MPa to 7.16 MPa, with the reference specimen having a strength of 6.85 MPa. The highest bending strength was obtained from the 2.0/S/N specimen. Among the specimens with narrow mesh aperture, the lowest strength was observed in the 4.0/D/N specimen, with a value of 5.03 MPa. Similarly, the lowest strength among single-layer mesh specimens was found in those containing 4 mm thick fibers. In double-layer mesh specimens, the highest value was again obtained with 2 mm thick fibers, consistent with the single-layer results.

For specimens with wide aperture, the highest values in both single-layer and double-layer configurations were also obtained with 2 mm thick fibers (7.13 MPa and 6.6 MPa, respectively). The lowest values for both configurations were recorded with 4 mm thick fibers (5.77 MPa and 4.93 MPa, respectively).

The general trend observed among the specimens indicates that increasing fiber thickness consistently leads to a reduction in bending strength. All double-layer mesh specimens exhibited lower strength values than the reference specimen. However, among the single-layer mesh specimens, both the narrow and wide mesh aperture configurations with 2 mm thick fibers achieved higher bending strength than the reference specimen. Specifically, the 2.0/S/N specimen showed a 4.5% increase, while the 2.0/S/W specimen demonstrated a 4.1% increase compared to the reference. The largest reduction in bending strength compared to the reference specimen was 20.3% in single-layer configurations and 28% in double-layer configurations. Additionally, double-layer mesh specimens consistently exhibited lower strength values than their single-layer counterparts. The strength differences between single-layer and double-layer configurations ranged from 4.6% to 14.6%. On the other hand, the effect of mesh spacing (narrow or wide) on bending strength did not exhibit a clear trend.

**Figure 18:**
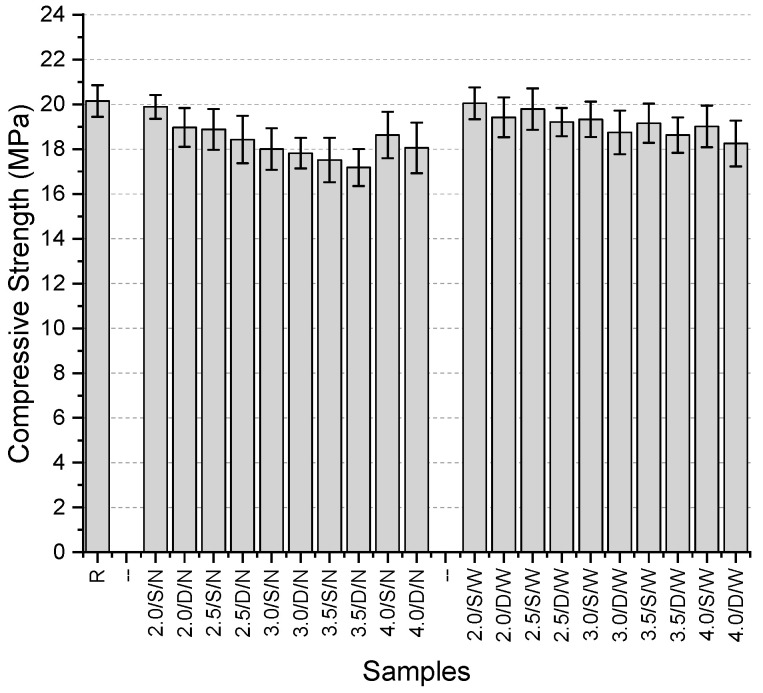
Compressive strength values of third preliminary trials.

The compressive strength values of the specimens (Figure 18) range from 17.18 MPa to 20.15 MPa, with the highest value obtained from the reference specimen. The maximum reduction in compressive strength due to fiber usage was recorded as 14.7%.

For specimens with narrow mesh aperture, the highest compressive strength was observed in those containing 2 mm thick fibers, both in single-layer (19.89 MPa) and double-layer (18.97 MPa) configurations. The lowest values for both configurations were found in specimens with 3.5 mm thick fibers. In these narrow mesh specimens, compressive strength decreased consistently as fiber thickness increased up to 4 mm, after which a slight recovery in strength was noted.

For specimens with wide mesh aperture, the highest compressive strength values were again obtained with 2 mm thick fibers, in both single-layer (20.05 MPa) and double-layer (19.42 MPa) configurations. The lowest values were observed in specimens containing 4 mm thick fibers (19.01 MPa for single-layer and 18.25 MPa for double-layer). Unlike the narrow mesh specimens, all wide mesh specimens showed a consistent decrease in compressive strength with increasing fiber thickness.

Across all specimens, the use of a double-layer mesh resulted in a reduction in compressive strength, with the decrease ranging from 1.1% to 4.6%. However, the use of wide mesh spacing led to an increase in compressive strength compared to all narrow mesh specimens. This increase ranged between 0.8% and 9.4%.

Although 2 mm thickness yielded the most favorable results in the second stage, the third stage included thicker (4 mm) double-layer configurations to investigate whether increased mesh volume or alternative spatial distribution could mitigate performance losses. This approach aimed to explore the possibility of offsetting bonding limitations through increased reinforcement presence. However, the findings indicated that the benefits of increased volume were not sufficient to counteract the negative effects of excessive thickness.

## 4. Conclusions

This study explored the feasibility of using 3D-printed PLA meshes as reinforcement in gypsum composites. The experimental findings indicate that:

- The addition of PLA meshes had minimal influence on bulk density values, with variations remaining within a narrow range. However, UPV values consistently decreased with mesh incorporation, suggesting a potential increase in void content or weaker fiber–matrix bonding.
- Contrary to expectations, most specimens incorporating PLA meshes exhibited lower bending and compressive strength compared to the reference sample. The maximum strength loss in bending and compressive tests reached 20.3% and 14.7%, respectively. Although this study did not include a detailed fracture analysis, it is recognized that mesh geometry—particularly aperture size and thickness—may lead to local stress concentrations, potentially acting as crack initiators under load. In some cases, excessively thick or tightly spaced mesh patterns may even compromise the composite’s integrity. Future research should include experimental or numerical investigation of failure mechanics to better understand these effects.
- Among the tested configurations, 2 mm thick fibers yielded relatively better results in bending strength, especially when used in a narrow mesh pattern. However, increasing fiber thickness beyond 2 mm generally resulted in diminished mechanical properties.
- Double-layer mesh configurations consistently resulted in lower strength values compared to single-layer applications, suggesting that excessive fiber content might hinder effective load transfer or introduce additional voids.
- No definitive trend was observed regarding the effect of narrow or wide mesh spacing on mechanical performance. However, some specimens with narrow aperture spacing exhibited slightly higher strengths.

The three stages of preliminary experiments were designed to iteratively explore and refine mesh parameters. In the first trial, very thin fibers (up to 0.16 mm) and large aperture sizes were tested, but poor mechanical performance was observed, likely due to insufficient material volume and inadequate bonding. The second trial focused on increasing fiber thickness (up to 4.0 mm), which initially improved compressive strength, though excessive thickness led to negative effects, such as poor embedding. In the third trial, selected mesh configurations with varying thicknesses were re-tested in single- and double-layer arrangements. This phase confirmed that intermediate fiber thicknesses (~2 mm) and single-layer structures provided more favorable performance. However, the use of double-layer meshes generally resulted in reduced strength, likely due to localized weaknesses introduced during casting.

It should be noted that no microstructural or interfacial tests (e.g., SEM or pull-out strength measurements) were conducted as part of this preliminary study. Future research is encouraged to investigate the bonding interface more deeply using such methods to better characterize the mechanical interaction between PLA meshes and gypsum. Given the inherent hydrophobicity and low surface roughness of PLA, bonding with gypsum matrix is likely to be suboptimal in the absence of surface treatment. In this study, PLA meshes were used without any surface modification. Future research should consider surface treatment techniques or chemical coupling agents to enhance interface adhesion, as well as methods for quantitatively characterizing bond performance.

Although most configurations tested in this preliminary study did not outperform the plain gypsum specimens, the work demonstrates the feasibility of using 3D printing to produce customized mesh structures for future composite reinforcement. Further optimization in terms of material properties, mesh design, and surface treatment may lead to improved outcomes in subsequent investigations.

## Figures and Tables

**Table 1:** Technical properties of gypsum.

Compound	Amount (%)
MgO	0.58
CaO	40.84
SO_3_	51.85
SiO_2_	3.27
Al_2_O_3_	0.36
Fe_2_O_3_	0.41
K_2_O	0.11
Na_2_O	0.12
Loss on ignition	18.95
**Physical properties**	
Density (g/cm^3^)	2.28
Fineness (m^2^/kg)	492

**Table 2:** Technical properties of PLA filament.

Property	Value
Density (g/cm^3^)	1.24
Tensile strength (MPa)	33.72
Bending strength (MPa)	76.12
Charpy Impact Strength (J/m^2^)	6.89
Printing Temperature (°C)	190–230
Filament diameter (mm)	1.75
Product mass (kg)	1

**Table 3:** Mesh types and samples codes used in the first preliminary trial studies.

Sample Code	Fiber Thickness	Aperture Size	Number of Layers
R	-	-	-
0.16/S/N	0.16 mm	3 × 3 mm	Single
0.20/S/N	0.20 mm	3 × 3 mm	Single
0.32/S/N	0.32 mm	3 × 3 mm	Single
0.32/S/W	0.32 mm	6 × 6 mm	Single
0.40/S/N	0.40 mm	3 × 3 mm	Single
0.60/S/N	0.60 mm	3 × 3 mm	Single
0.16/D/N	0.16 mm	3 × 3 mm	Double
0.20/D/N	0.20 mm	3 × 3 mm	Double
0.32/D/W	0.32 mm	6 × 6 mm	Double

**Table 4:** Comparison of mechanical performance under S-N configuration with varying fiber thickness.

Sample Code	Fiber Thickness	Compressive Strength	Bending Strength
0.16/S/N	0.16 mm	17.66 MPa	6.63 MPa
0.20/S/N	0.20 mm	18.31 MPa	6.69 MPa
0.40/S/N	0.40 mm	18.63 MPa	6.41 MPa
0.60/S/N	0.60 mm	18.17 MPa	6.71 MPa

**Table 5:** Mesh types and samples codes used in the second preliminary trials.

Sample Code	Fiber Thickness	Aperture Size	Number of Layers
R	-	-	-
1.0/S/W	1.0 mm	6 × 6 mm	Single
1.5/S/W	1.5 mm	6 × 6 mm	Single
2.0/S/W	2.0 mm	6 × 6 mm	Single
2.5/S/W	2.5 mm	6 × 6 mm	Single
3.0/S/W	3.0 mm	6 × 6 mm	Single
4.0/S/W	4.0 mm	6 × 6 mm	Single
5.0/S/W	5.0 mm	6 × 6 mm	Single
1.0/S/N	1.0 mm	3 × 3 mm	Single
1.5/S/N	1.5 mm	3 × 3 mm	Single
2.0/S/N	2.0 mm	3 × 3 mm	Single
2.5/S/N	2.5 mm	3 × 3 mm	Single
3.0/S/N	3.0 mm	3 × 3 mm	Single
4.0/S/N	4.0 mm	3 × 3 mm	Single
5.0/S/N	5.0 mm	3 × 3 mm	Single

**Table 6:** Mesh types and sample codes used in the third preliminary trial studies.

Sample Code	Fiber Thickness	Aperture Size	Number of Layers
R	-	-	-
2.0/S/N	2.0 mm	3 × 3 mm	Single
2.0/D/N	2.0 mm	3 × 3 mm	Double
2.5/S/N	2.5 mm	3 × 3 mm	Single
2.5/D/N	2.5 mm	3 × 3 mm	Double
3.0/S/N	3.0 mm	3 × 3 mm	Single
3.0/D/N	3.0 mm	3 × 3 mm	Double
3.5/S/N	3.5 mm	3 × 3 mm	Single
3.5/D/N	3.5 mm	3 × 3 mm	Double
4.0/S/N	4.0 mm	3 × 3 mm	Single
4.0/D/N	4.0 mm	3 × 3 mm	Double
2.0/S/W	2.0 mm	6 × 6 mm	Single
2.0/D/W	2.0 mm	6 × 6 mm	Double
2.5/S/W	2.5 mm	6 × 6 mm	Single
2.5/D/W	2.5 mm	6 × 6 mm	Double
3.0/S/W	3.0 mm	6 × 6 mm	Single
3.0/D/W	3.0 mm	6 × 6 mm	Double
3.5/S/W	3.5 mm	6 × 6 mm	Single
3.5/D/W	3.5 mm	6 × 6 mm	Double
4.0/S/W	4.0 mm	6 × 6 mm	Single
4.0/D/W	4.0 mm	6 × 6 mm	Double

## Data Availability

The original contributions presented in this study are included in the article. Further inquiries can be directed to the corresponding author.

## Abbreviations

The following abbreviations are used in this manuscript:

PLA	Polylactic acid
UPV	Ultrasonic pulse velocity
3D	Three dimensions
AR	Alkali resistant
MOR	Modulus of rupture
FDM	Fused deposition modelling
SLA	Selective laser sintering
ABS	Acrylonitrile-butadiene-styrene
PC	Polycarbonate
PA	Polyamide
TS	Turkish standard
EN	European norms
ASTM	American society of testing materials
